# Extracorporeal membrane oxygenation experience in COVID-19 pandemic: Report of two cases

**DOI:** 10.14744/nci.2020.86094

**Published:** 2020-11-24

**Authors:** Duygu Demiriz Gulmez, Elvan Tekir Yilmaz, Meltem Karamustafa, Ismail Olgun Akkaya, Pinar Tekin

**Affiliations:** 1 Department of Anesthesiology and Reanimation, Giresun University Faculty of Medicine, Giresun, Turkiye; 2 Department of Anesthesiology and Reanimation, Prof. Dr. A. Ilhan Ozdemir Training and Research Hospital, Giresun, Turkiye; 3 Department of Cardiovascular Surgery, Prof. Dr. A. Ilhan Ozdemir Training and Research Hospital, Giresun, Turkiye

**Keywords:** Acute respiratory distress syndrome, coronavirus disease 2019, extracorporeal membrane oxygenation

## Abstract

The new coronavirus disease, in which 100,000 of people are infected in the world, appears in tables ranging from asymptomatic course to severe acute respiratory distress syndrome. Extracorporeal membrane oxygenation (ECMO) is one of the salvage treatments applied in intubated patients due to high mortality. However, since ECMO treatment is a complicated treatment, the gain loss rate should be determined well. We aimed to share two cases that we applied ECMO treatment in our clinic. Although we could not achieve a successful result, we believe that new experiences should be shared in order to better understand the place of ECMO in coronavirus disease 2019 treatment.

The new coronavirus disease 2019 (COVID-19) has become a pandemic that has deeply affected the world. Although most of the COVID-19 cases are asymptomatic or have mild symptoms, some of the cases show acute respiratory distress syndrome (ARDS) symptoms, and some require mechanical ventilation (MV). Mortality in patients with MV needs remains high despite all treatments [1]. Extracorporeal membrane oxygenation (ECMO) therapy is recommended in severe ARDS cases; however, its effectiveness for COVID-19 has not been elucidated yet. The mortality rate is 82% in the deliberate number of cases reported [1]. As it is a new virus, it is very valuable to share every experience about this virus. Thus, we wanted to share our ECMO experience in two cases with severe ARDS.

## CASE REPORTS

**Figure 1:**
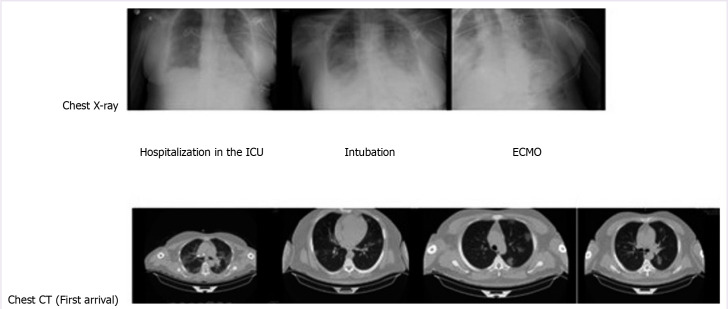
Chest X-ray and computed tomography images of a 76-year-old woman with COVID-19 infection.

**Case 1 –** A 76-year-old female patient was presented with only controlled hypertension disease. She had the symptoms of fever and cough. Due to the appearance of bilateral ground glass in her computed tomography (CT) scan, viral pneumonia was suspected and hydroxychloroquine sulfate, azithromycin, and oseltamivir were planned for 5 days. The first polymerase chain reaction (PCR) test was evaluated as SARS-CoV-2 positive. On the 2^nd^ day of the treatment, the patient’s condition worsened, and she was admitted into our intensive care unit, and high dose of vitamin C, immune plasma, and tocilizumab (a humanized monoclonal antibody against the interleukin-6 receptor) were added to the treatment. She was advised to lie in a prone position. On the 4^th^ day of hospitalization, she had deep hypoxia and the patient was intubated under elective conditions. Venovenous ECMO was planned for the patient, as the condition severe ARDS did not improve due to viral pneumonia despite all treatments with P/F ratio below 100 [2]. The chance of survival with a score of −1 was assessed as 57% [3]. Continuous renal replacement therapy was started on the patient who developed acute renal failure. Laboratory results and images are shown in Table 1 and Figure 1. However, the patient died on the 7^th^ day of ECMO treatment.

**Table 1:** Laboratory findings of the first case

	First arrival	Hospitalization in the ICU	Intubation	ECMO-1	ECMO-2	ECMO-3
WBC (10^3^/µL)	6,44	14.6	18	12	33	37,17
Lym (10^3^/µL)	0.94	0.5 109l	0,89	0,57	4,19	6,57
D-dimer (ng/mL)	2800	3959	3655	5079		
C-reactive protein (CRP) (mg/L)	129	246.18	214	192	453	571
Urea (mg/dL)	21	50	102	82	121	214
Creatinine (mg/dL)	0.48	0.53	1.8	0.49	0.48	0.81
ALT/AST (IU/L)	49/65	59/70	99/221	36/66	60/129	97/235
Ph	7.42	7.47	7.14	7.53	7.49	7.18
PO_2_ (mm Hg)	68	43.7	51	56	66	45
CO_2_ (mm Hg)	35	38.2	52	42	43	38
HCO_3_ (mmol/L)	24	27.2	15	35	32	17
Peep				12	10	12
P/F				56	73.3	45
ACT (sec)				205	200	215

ICU: Intensive care unit; ECMO: Extracorporeal membrane oxygenation; WBC: White blood cell; ALT: Alanine aminotransferase; AST: Aspartate aminotransferase.

**Figure 2:**
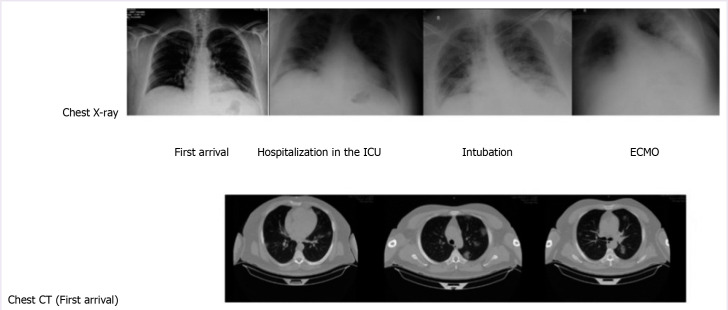
Chest X-ray and computed tomography images of a 48-year-old man with COVID-19 infection.

**Case 2 –** A 48-year-old male patient was admitted with the symptoms of fever and cough. Due to the appearance of bilateral ground glass on CT, viral pneumonia was suspected and hydroxychloroquine, oseltamivir oral for 5 days was planned. Bone marrow biopsy test was done due to a suspected malignancy with white blood cell count of 74X10^3^/µL. The first PCR test was negative, but the second test, which was taken after 48 h, was positive. However, during the follow-up period, the patient, whose oxygen saturation was 83% despite 5L/min through mask, was referred to our intensive care unit. The patient had increased respiratory difficulty and was taken under the support of MV. Rocuronium and demizolam infusion was initiated, and treatment with favipiravir, immunoglobulin, and imatinib was started for the patient whose biopsy was evaluated as chronic myeloid leukemia (CML). Venovenous ECMO treatment was planned on the 9^th^ day of intubation [2]. The ECMO procedure was started with 19Fr from the right internal jugular vein and 21 Fr canula from the right femoral vein. On the 7^th^ day of ECMO, the patient developed sudden hypotension, metabolic acidosis, and P/F rate quickly dropped below 100. Laboratory results and images are shown in Table 2 and Figure 2. The patient died despite all the necessary interventions.

**Table 2:** Laboratory findings of the second case

	First arrival	Hospitalization in the ICU	Intubation	ECMO-1	ECMO-2	ECMO-3
WBC (10^3^/µL)	74	66	70	55	59	60
Lym (10^3^/µL)	4,66	2,99	1,91	2,38	2,77	3,61
D-dimer (ng/mL)		249	262	4027		
C-reactive protein (CRP) (mg/L)	39	78	196.5	164.6	326	287
Urea (mg/dL)	30	25	27	55	69	82
Creatinine (mg/dL)	0.84	0.7	0.7	0.44	0.4	0.56
ALT/AST (IU/L)	16/27	22/57	33/70	42/54	23/54	25/63
Ph	7.48	7.45	7.21	7.62	7.5	7.53
PO_2_ (mm Hg)	72	63	32	44	94	55
CO_2_ (mm Hg)	25	31	54	33	39	43
HCO_3_ (mmol/L)	21	22	30	36	33	32
Peep				10	10	10
P/F				44	150	192
ACT (sec)				230	180	220

ICU: Intensive care unit; ECMO: Extracorporeal membrane oxygenation; WBC: White blood cell; ALT: Alanine aminotransferase; AST: Aspartate aminotransferase.

## DISCUSSION

Despite treatments such as antiviral drugs, steroids, antibody drugs, lung protective strategies, and prone position, patients who are intubated for COVID-19 have a high mortality rate. For this reason, salvage therapies come to mind for patients with severe ARDS as ECMO, but the treatment is expensive and complicated. For these reasons, it is necessary to be selective about the indication. ECMO indications are respiratory insufficiency, P/F ratio below 100, MV time of <7 days, and age below 65 years [3]. Some of the contraindications of ECMO therapy are anticoagulation, severe or multiple comorbidities, and MV for >14 days before ECMO initiation [2, 4]. In our first case, we were not successful due to the following reasons: Patient’s age, kidney failure, and increased need for inotropic support. In our second case, the patient was a newly diagnosed CML, but it was not among the definitive contraindications since cure could be achieved with treatment. The reason for our failure may be complications due to malignancy or ECMO-related hypercoagulopathies.

Taniguchi et al. [5] shared their reasons of success in the early ECMO treatment, current antiviral therapy, and continuous renal replacement therapy (CRRT). In our first case, we applied CRRT and current COVID-19 treatments, but time to start ECMO was delayed.

In the analysis of 32 ECMO cases shared by Jacobs et al. [6], 17 patients could not leave ECMO, ten patients died shortly after cannulation, five of them could be separated from ECMO and extubated, and one was discharged from the hospital. Two of these five people received tocilizumab with our first patient.

## Conclusion

Although the success of ECMO is not as promising as in H1N1, more clinical experience is needed to clarify its role in the treatment of SARS-CoV-2-related ARDS. It can be preferred as a salvage treatment method for patients, considering the gain-loss ratio when appropriate.
